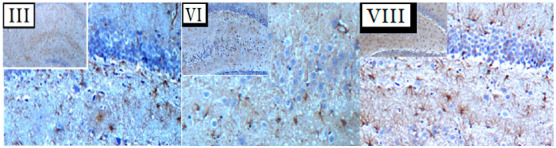# Correction: Al-Tawarah et al. *Rosmarinus officinalis* and *Mentha piperita* Oils Supplementation Enhances Memory in a Rat Model of Scopolamine-Induced Alzheimer’s Disease-like Condition. *Nutrients* 2023, *15*, 1547

**DOI:** 10.3390/nu17071238

**Published:** 2025-04-01

**Authors:** Nafe M. Al-Tawarah, Rawand H. Al-dmour, Maha N. Abu Hajleh, Khaled M. Khleifat, Moath Alqaraleh, Yousef M. Al-Saraireh, Ahmad Q. Jaradat, Emad A. S. Al-Dujaili

**Affiliations:** 1 Department of Medical Laboratory Sciences, Faculty of Science, Mutah University, Al-Karak 61710, Jordan; 2 Department of Cosmetic Science, Pharmacological and Diagnostic Research Centre, Faculty of Allied Medical Sciences, Al-Ahliyya Amman University, Amman 19328, Jordan; 3 Pharmacological and Diagnostic Research Center (PDRC), Faculty of Pharmacy, Al-Ahliyya Amman University, Amman 19328, Jordan; 4 Faculty of Medicine, Mutah University, Al-Karak 64710, Jordan; 5 Centre for Cardiovascular Science, Queen’s Medical Research Institute, University of Edinburgh, Edinburgh EH8 9YL, UK

In the original publication [1], there was a mistake in Figure 8. During paper submission, in Figure 8A, a mismatch error in the (4×–10×) coronal sections of three panels, at the III, VI, and VIII treatment levels, took place. The new corrected treatment panels—III, VI, and VIII—for Figure 8A appear below. The authors state that the scientific conclusions are unaffected by the above error. This correction was approved by the Academic Editor. The original publication has also been updated.